# Vision Measures in Aging Cohorts: Gaps and Opportunities

**DOI:** 10.1093/geroni/igaa057.2949

**Published:** 2020-12-16

**Authors:** Varshini Varadaraj, Ahmed Shakarchi, Lama Assi, Bonnielin Swenor

**Affiliations:** 1 Johns Hopkins University, Baltimore, Maryland, United States; 2 Johns Hopkins Wilmer Eye Institute, Baltimore, Maryland, United States

## Abstract

There is wide variation in the methods used to assess vision across existing aging cohort studies. While subjective vision measures are valuable in capturing individuals’ perspectives, objective criteria are often needed to fully characterize visual impairment in older adults. In addition, visual function extends beyond acuity; contrast sensitivity, visual fields, and depth perception are known to impact daily functioning, and are needed to comprehensively assess visual function. Broader uptake of subjective and objective vision measures, and harmonization of vision measures across datasets is needed to robustly examine the role of visual impairment on aging and health outcomes. In this presentation, we will draw on work using data from the National Health and Aging Trends Study, Health and Retirement Study, and Health, Aging and Body Composition Study, among others, to illustrate uses of vision data in aging cohort studies, and examine the gaps and opportunities for improvement in vision measures. Part of a symposium sponsored by Sensory Health Interest Group.

